# Supplementary material for: Inhibitory tone in the dentate gyrus dynamically prioritizes memory flexibility or stability by tuning a sensitivity-consistency continuum
Source: PLoS Biol. 2026 Aug 18;24(8):e3003956. doi: 10.1371/journal.pbio.3003956 (PMC13506102; doi:10.1371/journal.pbio.3003956)
Supplement: Supplementary file 1 [file pbio.3003956.s005.pdf]

Table S1

Fig.	Panel	Analysis/group	Statistic	Value	df	p value	η_p^2	1-β
1	b	PS	2-way ANOVA	F=31.26	2,15	<0.0001*	0.81	1
		Pre vs Post-CNO,		F=2.97	1,15	0.1050	0.16	0.36
		Sham (n=6)	*post hoc*$^{\$}$	t=0.22	15	0.9949		
		PV-Gi (n=6)	*post hoc*$^{\$}$	t=7.09	15	<0.0001*		
		PV-Gq (n=6)	*post hoc*$^{\$}$	t=3.88	15	0.0044*		
		Between groups,		F=2.51	2,15	0.1145	0.25	0.43
		Pre-CNO						
		Sham vs PV-Gi	*post hoc*$^{\$}$	t=0.13	30	0.9987		
		Sham vs PV-Gq	*post hoc*$^{\$}$	t=0.83	30	0.7941		
		PV-Gi vs PV-Gq	*post hoc*$^{\$}$	t=0.69	30	0.8685		
		Post-CNO						
		Sham vs PV-Gi	*post hoc*$^{\$}$	t=3.52	30	0.0042*		
		Sham vs PV-Gq	*post hoc*$^{\$}$	t=0.85	30	0.7823		
		PV-Gi vs PV-Gq	*post hoc*$^{\$}$	t=4.37	30	0.0004*		
	c	EPSP	2-way ANOVA	F=1.58	2,15	0.2382	0.17	0.30
		Pre vs Post-CNO,		F=0.87	1,15	0.3645	0.24	0.13
		Sham (n=6)	*post hoc*$^{\$}$	t=0.87	15	0.7789		
		PV-Gi (n=6)	*post hoc*$^{\$}$	t=1.52	15	0.3794		
		PV-Gq (n=6)	*post hoc*$^{\$}$	t=0.96	15	0.7248		
		Between groups,		F=2.25	2,15	0.1394	0.23	0.40
		Pre-CNO						
		Sham vs PV-Gi	*post hoc*$^{\$}$	t=1.83	30	0.2141		
		Sham vs PV-Gq	*post hoc*$^{\$}$	t=0.36	30	0.9779		
		PV-Gi vs PV-Gq	*post hoc*$^{\$}$	t=1.46	30	0.3917		
		Post-CNO						
		Sham vs PV-Gi	*post hoc*$^{\$}$	t=2.22	30	0.0977		
		Sham vs PV-Gq	*post hoc*$^{\$}$	t=0.66	30	0.8828		
		PV-Gi vs PV-Gq	*post hoc*$^{\$}$	t=1.56	30	0.3397		
	d	EPSP	2-way ANOVA	F=1.03	2,15	0.3792	0.12	0.23
		Pre vs Post-LTP,		F=102.8	1,15	<0.0001*	0.87	1
		Sham (n=6)	*post hoc*$^{\$}$	t=5.03	15	0.0004*		
		PV-Gi (n=6)	*post hoc*$^{\$}$	t=5.52	15	0.0002*		
		PV-Gq (n=6)	*post hoc*$^{\$}$	t=6.99	15	<0.0001*	0.20	0.36
		Between groups,		F=1.94	2,15	0.1777		
		Pre-LTP						
		Sham vs PV-Gi	*post hoc*$^{\$}$	t=1.65	30	0.2898		
		Sham vs PV-Gq	*post hoc*$^{\$}$	t=0.22	30	0.9947		
		PV-Gi vs PV-Gq	*post hoc*$^{\$}$	t=1.43	30	0.4108		
		Post-LTP						
		Sham vs PV-Gi	*post hoc*$^{\$}$	t=1.47	30	0.3888		
		Sham vs PV-Gq	*post hoc*$^{\$}$	t=0.51	30	0.9393		
		PV-Gi vs PV-Gq	*post hoc*$^{\$}$	t=1.99	30	0.1575		

$^{\$}$*Sidak post hoc comparison, alpha adjusted by Bonferroni at 0.05 significance level*
