# Supplementary material for: Inhibitory tone in the dentate gyrus dynamically prioritizes memory flexibility or stability by tuning a sensitivity-consistency continuum
Source: PLoS Biol. 2026 Aug 18;24(8):e3003956. doi: 10.1371/journal.pbio.3003956 (PMC13506102; doi:10.1371/journal.pbio.3003956)
Supplement: Supplementary file 2 [file pbio.3003956.s006.pdf]

Table S2

Fig.	Panel	Analysis/group	Statistic	Value	df	p value	η_p^2	1-β
2	b	Encoding	2-way ANOVA	F=12.31	2,35	<0.0001*	0.41	0.99
		Within groups,		F=55.86	1,35	<0.0001*	0.61	1
		Sham (n=13)	*post hoc*$	t=4.27	35	0.0004*		
		PV-Gi (n=13)	*post hoc*$	t=8	35	<0.0001*		
		PV-Gq (n=12)	*post hoc*$	t=0.81	35	0.8055		
		Between groups, Fam		F=6.58	2,35	0.0037*	0.27	0.89
		Sham vs PV-Gi	*post hoc*$	t=0.63	70	0.9999		
		PV-Gi vs PV-Gq	*post hoc*$	t=0.25	70	0.9918		
		Sham vs PV-Gq	*post hoc*$	t=0.19	70	0.9964		
		Test						
		Sham vs PV-Gi	*post hoc*$	t=3.29	70	0.0046*		
		PV-Gi vs PV-Gq	*post hoc*$	t=5.88	70	<0.0001*		
		Sham vs PV-Gq	*post hoc*$	t=2.65	70	0.02*		
	c	Consolidation	2-way ANOVA	F=0.04	2,39	0.9532	0.00	0.05
		Within groups,		F=60.23	1,39	<0.0001*	0.60	1
		Sham (n=13)	*post hoc*$	t=4.49	39	0.0002*		
		PV-Gi (n=17)	*post hoc*$	t=5.14	39	<0.0001*		
		PV-Gq (n=12)	*post hoc*$	t=3.95	39	0.001*		
		Between groups, Fam		F=3.97	2,39	0.0268*	0.16	0.68
		Sham vs PV-Gi	*post hoc*$	t=0.73	78	0.8486		
		PV-Gi vs PV-Gq	*post hoc*$	t=1.49	78	0.3636		
		Sham vs PV-Gq	*post hoc*$	t=2.07	78	0.1181		
		Test						
		Sham vs PV-Gi	*post hoc*$	t=0.73	78	0.8465		
		PV-Gi vs PV-Gq	*post hoc*$	t=1.07	78	0.6346		
		Sham vs PV-Gq	*post hoc*$	t=1.69	78	0.2587		
	d	Retrieval	2-way ANOVA	F=0.74	2,38	0.4798	0.03	0.17
		Within groups		F=38.16	1,38	<0.0001*	0.50	1
		Sham (n=14)	*post hoc*$	t=4.27	38	0.0004*		
		PV-Gi (n=14)	*post hoc*$	t=3.95	38	0.0010*		
		PV-Gq (n=13)	*post hoc*$	t=2.51	38	0.0485*		
		Between groups, Fam		F=1.121	2,38	0.3366	0.05	0.24
		Sham vs PV-Gi	*post hoc*$	t=0.98	76	0.6994		
		PV-Gi vs PV-Gq	*post hoc*$	t=1.31	76	0.4713		
		Sham vs PV-Gq	*post hoc*$	t=0.35	76	0.9787		
		Test						
		Sham vs PV-Gi	*post hoc*$	t=1.27	76	0.5000		
		PV-Gi vs PV-Gq	*post hoc*$	t=1.10	76	0.9994		
		Sham vs PV-Gq	*post hoc*$	t=1.14	76	0.5895		
	e	Control	2-way ANOVA	F=0.28	2,35	0.7571	0.01	0.09
		Within groups		F=29.28	1,35	<0.0001*	0.45	0.99
		Sham (n=13)	*post hoc*$	t=2.88	35	0.0196*		
		PV-Gi (n=13)	*post hoc*$	t=2.81	35	0.0235*		
		PV-Gq (n=12)	*post hoc*$	t=3.65	35	0.0026*		
		Between groups, Fam		F=1.05	2,35	0.3577	0.05	0.21
		Sham vs PV-Gi	*post hoc*$	t=0.14	70	0.9985		

		PV-Gi vs PV-Gq	*post hoc*[$]	t=1.24	70	0.5194		
		Sham vs PV-Gq	*post hoc*[$]	t=1.38	70	0.4265		
		Test						
		Sham vs PV-Gi	*post hoc*[$]	t=0.22	70	0.9947		
		PV-Gi vs PV-Gq	*post hoc*[$]	t=0.24	70	0.9931		
		Sham vs PV-Gq	*post hoc*[$]	t=0.45	70	0.9566		
	f	Encoding – 20cm	2-way ANOVA	F=0.95	2,18	0.4047	0.09	0.19
		Within groups,		F=49.79	1,18	<0.0001*	0.73	1
		Sham (n=6)	*post hoc*[$]	t=2.70	18	0.0429*		
		PV-Gi (n=8)	*post hoc*[$]	t=5.22	18	0.0002*		
		PV-Gq (n=7)	*post hoc*[$]	t=4.11	18	00019*		
		Between groups, Fam		F=0.56	2,18	0.5801	0.05	0.13
		Sham vs PV-Gi	*post hoc*[$]	t=0.23	36	0.9935		
		PV-Gi vs PV-Gq	*post hoc*[$]	t=0.37	36	0.9747		
		Sham vs PV-Gq	*post hoc*[$]	t=0.58	36	0.9166		
		Test						
		Sham vs PV-Gi	*post hoc*[$]	t=1.52	36	0.3580		
		PV-Gi vs PV-Gq	*post hoc*[$]	t=1.09	36	0.6256		
		Sham vs PV-Gq	*post hoc*[$]	t=0.45	36	0.9582		
	h	Sequential NOL	2-way ANOVA	F=7.02	2,14	0.0077*	0.50	0.94
		Between groups (n=8)		F=1.80	1,7	0.2208	0.20	0.20
		Fam	*post hoc*[$]	t=0.90	14	0.7648		
		10 cm	*post hoc*[$]	t=4.08	14	0.0034*		
		15 cm	*post hoc*[$]	t=0.02	14	>0.9999		
		Within groups (n=8)		F=14.48	2,14	0.0004*	0.67	0.99
		Saline Fam vs t1	*post hoc*[$]	t=0.2	14	0.9744		
		Saline Fam vs t2	*post hoc*[$]	t=3.57	14	0.0050*		
		Saline t1 vs t2	*post hoc*[$]	t=3.36	14	0.0076*		
		CNO Fam vs t1	*post hoc*[$]	t=4.85	14	0.0004*		
		CNO Fam vs t2	*post hoc*[$]	t=4.43	14	0.0009*		
		CNO t1 vs t2	*post hoc*[$]	t=0.42	14	0.8950		
4	b	Between groups (n=7 per group)	1-way ANOVA	F=6.65	2,18	0.0068*	0.42	0.89
		Sham vs PV-Gi	*post hoc*[$$]	q=3.58	18	0.0041*		
		Sham vs PV-Gq	*post hoc*[$$]	q=2.40	18	0.0494*		
	d	Between groups (n=7 per group)	1-way ANOVA	F=3.80	2,18	0.0420*	0.29	0.61
		Sham vs PV-Gi	*post hoc*[$$]	q=2.53	18	0.0384*		
		Sham vs PV-Gq	*post hoc*[$$]	q=2.21	18	0.0719		

[$] *Sidak post hoc comparison, alpha adjusted by Bonferroni at 0.05 significance level*

[$$] *Dunnett's multiple comparison test*
