# Supplementary material for: Inhibitory tone in the dentate gyrus dynamically prioritizes memory flexibility or stability by tuning a sensitivity-consistency continuum
Source: PLoS Biol. 2026 Aug 18;24(8):e3003956. doi: 10.1371/journal.pbio.3003956 (PMC13506102; doi:10.1371/journal.pbio.3003956)
Supplement: Supplementary file 3 [file pbio.3003956.s007.pdf]

**Table S3**

| Fig. | Panel | Analysis/group | Statistics | Value | df | p value | $\eta_p^2$ | 1-β |
|---|---|---|---|---|---|---|---|---|
| S2 | c | Disynaptic EPSP | 2-way ANOVA | F=1.71 | 2,13 | 0.2192 | 0.20 | 0.31 |
| | | Pre vs post-CNO, | | F=0.68 | 1,13 | 0.4227 | 0.05 | 0.12 |
| | | Sham (n=6) | *post hoc*[$] | t=1.89 | 13 | 0.2213 | | |
| | | PV-Gi (n=5) | *post hoc*[$] | t=0.76 | 13 | 0.8421 | | |
| | | PV-Gq (n=5) | *post hoc*[$] | t=0.42 | 13 | 0.9671 | | |
| | | Between groups, Pre-CNO | | F=0.41 | 2,13 | 0.6667 | 0.05 | 0.13 |
| | | Sham vs PV-Gi | *post hoc*[$] | t=1.01 | 26 | 0.6841 | | |
| | | Sham vs PV-Gq | *post hoc*[$] | t=1.03 | 26 | 0.6692 | | |
| | | PV-Gi vs PV-Gq | *post hoc*[$] | t=0.02 | 26 | >0.9999 | | |
| | | Post-CNO | | | | | | |
| | | Sham vs PV-Gi | *post hoc*[$] | t=0.28 | 26 | 0.9895 | | |
| | | Sham vs PV-Gq | *post hoc*[$] | t=0.65 | 26 | 0.8891 | | |
| | | PV-Gi vs PV-Gq | *post hoc*[$] | t=0.35 | 26 | 0.9792 | | |
| | d | Trisynaptic EPSP | 2-way ANOVA | F=116.4 | 2,15 | <0.0001[*] | 0.94 | 1 |
| | | Pre vs post-CNO, | | F=93.69 | 1,15 | <0.0001[*] | 0.86 | 1 |
| | | Sham (n=6) | *post hoc*[$] | t=2.18 | 15 | 0.1301 | | |
| | | PV-Gi (n=6) | *post hoc*[$] | t=17.67 | 15 | <0.0001[*] | | |
| | | PV-Gq (n=6) | *post hoc*[$] | t=3.08 | 15 | 0.0224[*] | | |
| | | Between groups, Pre-CNO | | F=15.74 | 2,15 | 0.0002[*] | 0.67 | 0.99 |
| | | Sham vs PV-Gi | *post hoc*[$] | t=0.48 | 30 | 0.9496 | | |
| | | Sham vs PV-Gq | *post hoc*[$] | t=1.32 | 30 | 0.4808 | | |
| | | PV-Gi vs PV-Gq | *post hoc*[$] | t=1.80 | 30 | 0.2231 | | |
| | | Post-CNO | | | | | | |
| | | Sham vs PV-Gi | *post hoc*[$] | t=8.95 | 30 | <0.0001[*] | | |
| | | Sham vs PV-Gq | *post hoc*[$] | t=1.89 | 30 | 0.1914 | | |
| | | PV-Gi vs PV-Gq | *post hoc*[$] | t=10.85 | 30 | <0.0001[*] | | |
| S3 | c | cFos in PV+ cells (n=8,8,6) | 1-way ANOVA | F=149.9 | 2.19 | <0.0001[*] | 0.48 | 0.95 |
| | | Sham vs PV-Gi | *post hoc*[$] | t=5.89 | 19 | <0.0001[*] | | |
| | | Sham vs PV-Gq | *post hoc*[$] | t=11.7 | 19 | <0.0001[*] | | |
| | | PV-Gi vs PV-Gq | *post hoc*[$] | t=17.16 | 19 | <0.0001[*] | | |
| | d | Home-cage (n=2) vs Exploration (n=8) | t-test independent measures | t=3.41 | 8 | 0.009[*] | 0.59 | 0.1 |
| S4 | c | Habituation 1 | 2-way ANOVA | F=1.32 | 2,68 | 0.2732 | - | - |
| | | *Between groups* | | F=3.29 | 2,68 | 0.0430[#] | 0.08[#] | 0.41[#] |
| | | Habituation 2 | | F=0.02 | 2,68 | 0.9792 | - | - |
| | | Fam (CNO) | | F=0.03 | 2,68 | 0.9653 | - | - |
| | | Test (CNO) | | F=1.34 | 2,68 | 0.2674 | - | - |
| | | Fam (Saline) | | F=1.57 | 2,68 | 0.2146 | - | - |
| | | Test (Saline) | | F=1.72 | 2,68 | 0.1858 | - | - |
| | e | Time in zone | 2-way ANOVA | F=0.0003 | 2.114 | >0.999 | - | - |
| | | Distance travelled | | F=0.005 | 2.152 | 0.99 | - | - |
| | | Resting time | | F=0.052 | 2.114 | 0.95 | - | - |
| | | Entries in zone | | F=0.1 | 2.102 | 0.89 | - | - |

[$]*Sidak post hoc comparison, alpha adjusted by Bonferroni at 0.05 significance level*

[#]*Statistically significant difference but low partial eta square so that its confidence is low to accept alternative hypothesis*
