# Supplementary material for: Inhibitory tone in the dentate gyrus dynamically prioritizes memory flexibility or stability by tuning a sensitivity-consistency continuum
Source: PLoS Biol. 2026 Aug 18;24(8):e3003956. doi: 10.1371/journal.pbio.3003956 (PMC13506102; doi:10.1371/journal.pbio.3003956)
Supplement: Supplementary file 4 [file pbio.3003956.s011.docx]

**REVIEWS:

Reviewer #1: Pérez-Montoyo et al., describe a series of physiological, behavioral, and computational experiments to elucidate the role of hippocampal parvalbumin-expressing interneuron in object-location learning. The authors focus on an important question and have made a substantial effort to address it experimentally. Unfortunately, there are several limitations in the interpretability of their data, in part due to inherent technical limitations of the approach and the experimental design. There are also several key conclusions which are not sufficiently supported by the data presented by the authors in my opinion.**

**Major points:**

**1. As shown on Figure S1, the viral constructs used by the authors affect PV interneurons in both the DG and CA3. This problem is not unique to their study (see e.g. Aery Jones et al., 2021, Cell Reports) but the assumption that the manipulation affects predominantly the DG is the only thing that links the experiment to the model here and is therefore critical.**

We thank the reviewer for raising this important point. We agree that the viral manipulation was not strictly confined to the DG and also affected PV+ interneurons in CA3, as reported in related studies using comparable strategies. We have therefore clarified this anatomical limitation in the revised manuscript.

Although the manipulation was not DG-exclusive, the anatomical distribution of infected PV+ neurons indicated a clear predominance of DG targeting, with much stronger infection in DG than in CA3 or CA1 (DG: 93 ± 3%; CA3: 33 ± 5%; CA1: 5 ± 2%). Thus, while we cannot claim that the manipulation was restricted to the DG, the viral strategy predominantly affected this hippocampal subregion.

Importantly, our electrophysiological data provide functional support for the interpretation that the main effect arises from modulation of the DG relay. In anesthetized mice, manipulation of PV+ interneurons altered the propagation of activity through the trisynaptic pathway (EC → DG → CA3 → CA1), whereas transmission through the disynaptic pathway bypassing the DG (EC → CA3 → CA1) remained largely unchanged. These findings indicate that the effects observed downstream in CA1 are more closely associated with altered DG-dependent processing than with a direct modification of CA3 network transmission. We have now included these data as Supplementary Figure S2 and added the corresponding description to the Results section.

Most importantly, this consideration does not alter the validity of the main finding or the conceptual conclusion of the study. Our central result is that PV-mediated inhibition during encoding controls how hippocampal-dependent object–location representations are formed and later expressed behaviorally. The anatomical spread to CA3 limits a strictly DG-exclusive interpretation of the viral manipulation, but it does not undermine the conclusion that altering PV-mediated inhibitory control within the DG–CA3 hippocampal circuit shifts the balance between sensitivity and consistency of memory representations. Consistent with this view, the computational model was not intended to depend on a perfectly DG-restricted manipulation, but to test how changes in PV-mediated inhibition influence the formation, discrimination, and consistency of hippocampal-like memory representations.

We have revised the manuscript to make this point explicit. We now acknowledge that a contribution from CA3 PV+ interneurons cannot be fully excluded, while also clarifying that the anatomical predominance of DG targeting, the pathway-specific electrophysiological evidence, and the behavioral/modeling convergence support the main conclusion that PV-mediated inhibition regulates the sensitivity-consistency balance during memory encoding.

In line with this, we have changed a sentence in the Results section of the model to avoid potential confusion and to better reflect the generality of the approach:

*“To simulate the inhibitory control exerted by PV^+^ interneurons in the DG” -> “To simulate PV-mediated inhibitory control within the hippocampal network”.*

And we have also added the reference to the new data in the Results section:

*“Although viral expression was not strictly restricted to the DG, infected PV^+^ neurons were predominantly located in this region, with lower levels of infection observed in CA3 and minimal infection in CA1 (DG: 93 ± 3%; CA3: 33 ± 5%; CA1: 5 ± 2%). Thus, the manipulation primarily targeted DG PV^+^ interneurons, although a contribution from CA3 PV^+^ interneurons cannot be excluded.*

*To assess the functional impact of this manipulation on hippocampal circuit activity, we compared responses through the trisynaptic pathway (EC → DG → CA3 → CA1) and the disynaptic pathway bypassing the DG (EC → CA3 → CA1) in anesthetized mice. PV^+^ interneuron manipulation altered activity propagation through the trisynaptic pathway, whereas transmission through the disynaptic pathway remained largely unchanged. This pathway-specific effect supports the interpretation that the manipulation mainly affected DG-dependent hippocampal processing, rather than producing a generalized alteration of CA3-driven transmission to CA1.”*

Moreover, we have also added a paragraph in the Discussion:

*“A methodological consideration is that the viral manipulation was not strictly confined to the DG and also affected PV^+^ interneurons in CA3. Thus, our results should not be interpreted as arising from an exclusively DG-restricted manipulation. Nevertheless, this limitation does not alter the main conclusion of the study. Anatomically, viral expression was strongly predominant in the DG, with lower infection in CA3 and minimal infection in CA1. Functionally, PV^+^ interneuron manipulation altered activity propagation through the DG-dependent trisynaptic pathway, whereas transmission through the disynaptic pathway bypassing the DG remained largely unchanged. These observations support the interpretation that the behavioral effects are primarily linked to altered DG-dependent processing, while acknowledging that a contribution from CA3 PV^+^ interneurons cannot be fully excluded. Importantly, the central concept proposed here does not require an absolutely DG-exclusive manipulation. Rather, our findings indicate that PV-mediated inhibitory control within the hippocampal DG–CA3 circuit shapes the initial encoding of object–location representations and regulates the balance between sensitivity and consistency. The computational model formalizes this principle at the network level by testing how changes in inhibitory tone influence the formation, discrimination, and consistency of hippocampal-like memory representations. Thus, the anatomical spread of the viral manipulation limits the spatial specificity of the interpretation, but does not undermine the validity of the behavioral finding or the proposed sensitivity–consistency framework.*”

**2. There are several assumptions inherent to the model about how sparsity or overlap of granule cell activity relates to performance in discrimination tasks that have no link to the experiments. The bare minimum the authors could do is to quantify the cfos expression in GCs in addition to the cfos expression in PV interneurons, similar to e.g. Stefanelli et al., 2016, Neuron (which should also be discussed as they perform extremely similar experiments) or Chawla et al., 2005, Hippocampus. A measure of GC activity through calcium imaging or electrophysiology during interneuron manipulations could decisively strengthen a potential link to the model and help discriminate effects of granule cells from those of mossy cells and CA3 pyramidal cells (see also point 1).**

We thank the reviewer for this important comment. We agree that GC recruitment is relevant to the interpretation of DG computations, but we would like to clarify that the computational model does not depend on a direct quantitative mapping between total GC activity, sparsity, or overlap and behavioral performance.

In response to the reviewer’s suggestion, we present the quantification of cFos expression in DG GCs following memory encoding. Consistent with previous work using related manipulations of DG PV+ interneurons (Stefanelli et al, 2016), we did not observe significant differences in the overall number of cFos-positive GCs across experimental groups. We provide these data in Fig. R1 for the reviewer’s consideration.

This result should be interpreted in light of what cFos reports, particularly in dentate granule cells. cFos is not a direct readout of neuronal firing or instantaneous activity; rather, it reflects the engagement of intracellular signaling pathways, including NMDA-dependent calcium signaling, that lead to activity-dependent transcription over a delayed temporal window. Thus, cFos expression depends on the intensity, duration, synaptic nature, and intracellular consequences of neuronal activation, rather than on spiking per se (Barros et al., 2015). Other immediate early genes, like the one indicated by the reviewer (Arc; Chawla et al., 2005) may have other expression profiles.

This distinction is especially important for GCs. Unlike hippocampal CA1 pyramidal neurons, in which cFos can more reliably report repeated neuronal recruitment, DG GCs show sparse cFos activation and a marked regulation of cFos re-expression. In particular, repeated behavioral activation can induce ΔFosB-dependent repression of cFos in DG granule cells, whereas CA1 neurons remain more permissive to repeated cFos expression (Barros et al., 2015; Lamothe-Molina et al., 2022). Therefore, similar numbers of cFos-positive GCs across groups do not exclude differences in which GCs are recruited, how different input patterns are represented, or how consistently these representations are maintained. Global cFos quantification provides a useful control for large changes in GC recruitment, but it does not directly measure the ensemble-specific or representational features that are most relevant to the model.

We have clarified the level of abstraction of the model in the revised manuscript. The model is not intended as a cell-by-cell reconstruction of DG microcircuitry, nor does it require total GC cFos expression to predict behavioral performance. Instead, it formalizes how changes in PV-mediated inhibitory control can alter population-level dynamics, affecting the sensitivity and consistency of hippocampal-like representations. GC sparsity and ensemble overlap are possible biological implementations of these dynamics, but they are not independently constrained by total cFos counts.

We have clarified the level of abstraction of the model in the revised manuscript. The model is not intended as a cell-by-cell reconstruction of DG microcircuitry, nor does it require total GC cFos expression to predict behavioral performance. Instead, it formalizes how changes in PV-mediated inhibitory control can alter population-level dynamics, affecting the sensitivity and consistency of hippocampal-like representations. GC sparsity and ensemble overlap are possible biological implementations of these dynamics, but they are not independently constrained by total cFos counts. Thus, a similar total number of cFos-positive GCs can coexist with differences in how individual memory representations are organized, separated, and maintained within the network.

We agree that calcium imaging or large-scale electrophysiological recordings during behavior would provide a more direct assessment of GC ensemble recruitment, representational overlap, and temporal dynamics during PV+ interneuron manipulation. Such experiments would be valuable in future studies, but they are beyond the scope of the present work.

Finally, as discussed in response to point 1, our *in vivo* electrophysiological data show that PV+ interneuron manipulation altered propagation through the trisynaptic pathway (EC → DG → CA3 → CA1), while leaving transmission through the DG-bypassing disynaptic pathway (EC → CA3 → CA1) largely unchanged. This supports the view that the manipulation primarily affected DG-dependent hippocampal processing, while acknowledging that a contribution from CA3 PV+ interneurons cannot be fully excluded.

**Figure R1. cFos expression in the DG induced by context exploration. (a)** Representative microphotographs of the DG showing immunostained cFos+ (green) and infected PV^+^ cells (red). Scale bar: 100µm. **(b)** Quantification of cFos+ cells in the DG, excluding double PV^+^ and cFos+ cells (white arrows in a), showing no statistical differences between Sham (n=8), PV-Gi (n=9) and PV-Gq (n=6) groups (One-way ANOVA: F(2,20)=1.93, p=0.171).

Stefanelli T, Bertollini C, Lüscher C, Muller D, Mendez P (2016). Hippocampal somatostatin interneurons control the size of neuronal memory ensembles. *Neuron* 89:1074-1085. doi: [10.1016/j.neuron.2016.01.024](https://doi.org/10.1016/j.neuron.2016.01.024)

Barros VN, Mundim M, Galindo LT, Bittencourt S, Porcionatto M and Mello LE (2015) The pattern of c-Fos expression and its refractory period in the brain of rats and monkeys. *Front. Cell. Neurosci*. 9:72. doi: [10.3389/fncel.2015.00072](https://doi.org/10.3389/fncel.2015.00072)

Chawla MK, Guzowski JF, Ramirez-Amaya V, et al. Sparse, environmentally selective expression of Arc RNA in the upper blade of the rodent fascia dentata by brief spatial experience. *Hippocampus*. 2005;15(5):579-586. doi:[10.1002/hipo.20091](https://doi.org/10.1002/hipo.20091)

Lamothe-Molina PJ, Franzelin A, Beck L, et al (2022). ΔFosB accumulation in hippocampal granule cells drives cFos pattern separation during spatial learning. *Nat Commun*. 13(1):6376. doi:[10.1038/s41467-022-33947-w](https://www.nature.com/articles/s41467-022-33947-w)

**3. On page 4, line 3, the authors claim that "the impaired performance observed in PV-Gq animals likely reflects a reduction in discrimination capacity rather than a general deficit in spatial memory encoding". While stated here as a possibility, the authors later use this as a central tenet of their manuscript without ever backing it with direct evidence. To support this claim, they should show whether a version of this task with a larger displacement or a shorter interval to testing can be learned by the animals to show that the deficit is not related to a global impairment of spatial memory or decreased stability of a formed memory trace.**

We thank the reviewer for this important suggestion. To directly address this concern, we performed an additional NOL experiment using a larger object displacement (20 cm) while maintaining the same 24 h retention interval and PV manipulation during encoding.

Under these conditions, control, PV-Gi, and PV-Gq animals all successfully discriminated the displaced object, indicating preserved spatial memory formation and retrieval in PV-Gq mice when task demands are reduced. These findings further support the interpretation that the impairment observed in PV-Gq animals in the standard NOL paradigm, using a 15 cm displacement, reflects not a general deficit in spatial memory encoding or stability, but rather a reduced ability to discriminate subtle spatial changes.

We have incorporated these new data into the revised manuscript (Fig.2, new panel f) and modified the corresponding text to clarify this interpretation.

**4. The outcome of the task in Fig 2f,g is ambiguous: One can equally well argue that the increased interest in the displaced object on the second trial on day 2 reflects learning of the 5 cm displacement compared to the first trial on that day one minute prior. The better comparator would be the original NOL task with 15 cm displacement and 24 hour interval.**

We agree that repeated testing could, in principle, raise the possibility that animals compare each displacement condition to the immediately preceding one. However, under the current experimental conditions, the presented results (Fig. 2h), and additional results shown here for review purposes (see below Fig. R2), argue against this interpretation.

First, the chemogenetic manipulation was restricted to the encoding phase, and therefore no CNO was administered during the recall session. Thus, any within-session updating or short-term comparison processes occurring across successive displacement trials would be expected to affect both conditions similarly. Despite this, PV-Gi animals injected with CNO reliably detected the initial 10 cm displacement, whereas when the same animals were injected with saline they only showed significant discrimination after the additional 5 cm displacement.

Second, we performed an additional experiment in control animals to assess short-term (STM) memory. In this experiment, animals (n=9) were habituated to the arena across two consecutive days. On day 3, the animals performed the encoding and test sessions consecutively, with an interval of 1 min between sessions. During testing, one object was displaced by 5 cm. Under these conditions, animals did not show a significant preference for the displaced object, with DI comparable between familiarization and test sessions. We provide these data in Fig. R2 for the reviewer’s consideration.

Finally, the manuscript already includes the comparator requested by the reviewer, which we understand to be a standard NOL experiment using a single 15 cm object displacement after a 24 h delay (Fig. 2b-e). In this experiment, both control and PV-Gi animals successfully detected the displacement, with PV-Gi animals exhibiting significantly enhanced discrimination performance. The 15 cm displacement used in the standard NOL task is specified in the Methods section.

**Figure R2. Short-term memory (STM) NOL. (a)** Temporal structure of the task: encoding (familiarization) and test sessions are performed within a 1-min interval. (b) Performance of control mice (WT, n=9) in both sessions, measured as the DI (paired t-test: t(8)=0.01, p=0.991). Data represent mean ± sem.

**5. For the task on Figure 4, it is unclear whether this reflects a deficit in context discrimination or primarily of increased workload as claimed by the authors. To distinguish these possibilities, we would need to know how animals in the different groups perform in a standard contextual conditioning task with a 24 hour interval.**

We thank the reviewer for raising this important point. We agree that the multi-arena NOL task does not isolate context discrimination in the strict sense of a standard contextual conditioning paradigm. The task requires animals to maintain multiple object–context–location associations, and therefore combines contextual information with object identity, spatial location, and associative load/interference demands.

However, we think that a primary deficit in discrimination is unlikely to explain the observed impairment. First, reducing PV-mediated inhibition did not impair discrimination in the lower-load NOL experiments. On the contrary, PV-Gi animals showed enhanced fine spatial discrimination when the object displacement was subtle (Fig. 2). Thus, reduced inhibition does not appear to produce a generalized impairment in discrimination. Second, in the multi-arena task, the impairment in PV-Gi animals was not an all-or-none failure. When performance was examined across individual arenas, PV-Gi animals still showed discrimination in some contexts, but retained fewer contexts overall (Fig. 4). This indicates that their ability to use contextual and spatial information was not abolished *per se*. Rather, the deficit emerged when several object–context–location associations had to be maintained over the same retention interval. Last, previous work manipulating DG PV+ interneurons during contextual fear conditioning reported no detectable deficits in long-term contextual memory, despite effective activation of the PV+ interneuron population (Stefanelli et al, 2016). We therefore interpret the Fig. 4 phenotype as reduced memory consistency under high associative load/interference, rather than as a primary inability to discriminate contexts.

We agree that a standard contextual conditioning experiment with a 24 h interval could provide additional information about context learning in a broader sense. However, such a task would not be a direct control for the computation addressed here. Contextual fear conditioning relies on reinforced aversive learning and freezing as the main behavioral readout, engaging partially distinct mechanisms, including amygdalar and stress-related circuits. Similarly, place-preference paradigms would introduce appetitive/reward components and striatal/dopaminergic mechanisms. These paradigms would therefore test context memory under different motivational, behavioral and circuit conditions, rather than the spontaneous object-location memory processes examined in the present study.

We have added some text to the Discussion:

*“A limitation of the multi-arena NOL task is that it cannot fully dissociate impaired contextual discrimination from impaired maintenance of multiple object–context–location associations. However, several observations argue against a generalized deficit in contextual discrimination. First, PV-Gi animals showed enhanced fine spatial discrimination in the low-load sequential NOL task, indicating that reduced inhibition does not impair discrimination per se. Second, in the multi-arena NOL task, PV-Gi animals did not fail uniformly across all arenas, but showed above-threshold discrimination in some contexts while retaining fewer contexts overall. This suggests that their ability to use contextual and spatial information was not abolished, but that performance was compromised when multiple object–context–location associations had to be maintained. Similarly, PV-Gq animals showed intermediate performance in the multi-arena NOL task rather than a generalized impairment, indicating that increased inhibition also does not abolish contextual discrimination. Together, these observations argue against a simple deficit in contextual discrimination. This interpretation is also consistent with previous work showing that manipulation of DG PV^+^ interneurons during contextual fear conditioning did not produce detectable deficits in long-term contextual memory discrimination, despite effective activation of the PV^+^ interneuron population (Stefanelli et al, 2016).”*

Stefanelli T, Bertollini C, Lüscher C, Muller D, Mendez P (2016). Hippocampal somatostatin interneurons control the size of neuronal memory ensembles. *Neuron* 89:1074-1085.

**Minor points:**

**1. Figure 1d: There should be a comparison between the magnitude of LTP in the three groups. It appears that plasticity increases both with activation and inhibition of PV interneurons. It would be interesting to see if that difference is significant and I would encourage the authors to discuss it.**

We thank the reviewer for this suggestion. We have now directly compared the magnitude of LTP across the three experimental groups. Although both PV-Gi and PV-Gq animals showed visually increased potentiation relative to controls, these differences were not statistically significant (two-way ANOVA: *Interaction* F(2,15)=1.035, p=0.379; *pre-postLTP* F(1,15)=102.8, p>0.0001; *Group* F(2,15)=1.943, p=0.177). We have included this analysis in Table S1 and clarified the corresponding text in the Results section.

**2. Fig S1: Should also show the fraction of DREADD expressing cells that are PV positive to rule out leak expression in non-target cells.**

We have now quantified the specificity of viral expression by determining the fraction of PV-positive cells that expressed the DREADD. We found that 96.96 ± 1.13% of DREADD-expressing cells were PV+ cells, indicating highly specific targeting of the intended interneuron population. We have incorporated this data into the manuscript.

**Reviewer #2: The manuscript by Pérez-Montoyo, María Caramés et al tackles an interesting question of how the memory system achieves sensitivity and consistency. I found these results to be very counterintuitive at first - especially the idea that reducing inhibition increases discrimination - so definitely a provocative study! The authors did an excellent job of walking me through their results and I especially appreciated the results from the computational mode.**

**Please address the following:**

**For the NOL encoding result, could the authors also report the total time exploring objects during the encoding phase (sample phase)? In other words, please exclude the possibility that the DI differences between the 3 groups during testing is not a result of reduced exploration during the sample phase. I see in the methods that the sample phase was terminated after 20 s of exploration of both objects or after 10 min in the arena.**

We thank the reviewer for highlighting this important point. We agree that differences in object exploration during the encoding phase could potentially influence subsequent performance during retrieval and therefore represent a potential confounding factor.

To minimize this possibility, the NOL protocol was designed such that animals remained in the familiarization session until they accumulated 20 s of object exploration or until a maximum duration of 10 min was reached. Consequently, most animals explored the objects for the full 20 s, and only a small number failed to reach this criterion within the allocated time.

As requested by the reviewer, we quantified the total object exploration time during the encoding phase for all experimental groups. These data are now shown in Fig. R3 and have been incorporated into the revised manuscript. Importantly, no significant differences were observed among groups (Kruskal–Wallis test: H(2) = 1.073, p = 0.584), indicating that the differences observed in the DI during retrieval are unlikely to be explained by differences in object exploration during encoding.

**Figure R3. Exploration times during the encoding session in the NOL task under PV manipulation.** Total object exploration during the encoding (familiarization) phase did not differ between experimental groups (Sham: 19.98 ± 0.08 s; PV-Gi: 19.41 ± 0.61 s; PV-Gq: 18.94 ± 0.79 s). Data are shown as mean ± SEM.

**In 2G, it seems that the control is not able to perform the task for the 10 cm displacement. Please consider results in (**[**https://urldefense.com/v3/__https://elifesciences.org/articles/65786__;!!D9dNQwwGXtA!UOJbehEqxbWcizzH3iKIhK5W8kN0zSCJSCaMXOGNBpmVah-VPi4dOHkIqjvmNVHtutT7MeQMXd680w$**](https://urldefense.com/v3/__https:/elifesciences.org/articles/65786__;!!D9dNQwwGXtA!UOJbehEqxbWcizzH3iKIhK5W8kN0zSCJSCaMXOGNBpmVah-VPi4dOHkIqjvmNVHtutT7MeQMXd680w$) **), in which controls are sensitive to 10 cm displacement.**

We thank the reviewer for this helpful comment and for pointing us to this relevant study. We agree that Pofahl et al. reported discrimination of an approximately 9–10 cm object displacement in control animals. However, their task differs from ours in two important aspects. First, and most importantly, Pofahl et al. tested memory after a shorter inter-trial interval of 90 min, whereas we tested animals after 24 h to assess long-term memory. Second, their arena was smaller than ours, so the same absolute displacement likely represented a larger relative spatial change.

Thus, we do not interpret the lack of discrimination at 10 cm in our controls as evidence that control animals are generally unable to detect this displacement. Rather, under our experimental conditions, a 10 cm displacement may not be sufficient to support reliable long-term discrimination (in baseline conditions). Importantly, the two studies are conceptually consistent: both identify an intermediate displacement range that is sensitive to DG/GC function (around 9–10 cm in Pofahl et al. under short-delay conditions, and around 15 cm in our 24 h paradigm) whereas larger displacements, such as 20 cm in our study, become less sensitive to DG manipulation.

**Along these lines, while your results indicate that PV disinhibition promotes discrimination - they don't help explain why fine discrimination is dependent on the dentate gyrus. (In fact, you don't observe discrimination of 10 cm in the intact case). Could you add to your discussion and speculate on how PV disinhibition may be flexibly realized in the DG? It could be useful to consider recent data about dentate spikes during NOL memory (Dupret).**

We thank the reviewer for this insightful comment and for highlighting the importance of understanding how PV-mediated inhibition may be flexibly regulated in the DG. We agree that an important open question raised by our findings concerns the physiological mechanisms that allow dynamic modulation of inhibitory tone during memory processing.

As suggested by the reviewer, we have expanded the Discussion to consider circuit-level mechanisms that may support such flexibility. In particular, we now discuss how PV interneuron activity is embedded within a broader inhibitory network that includes disinhibitory interneuron classes such as VIP-expressing cells, as well as somatostatin (SOM)-positive interneurons, whose coordinated activity has been shown to depend on behavioral and network state. These mechanisms, together with neuromodulatory influences and activity-dependent plasticity within inhibitory circuits, may allow transient shifts in DG inhibitory tone that regulate the resolution of spatial representations.

Following the reviewer’s suggestion, we also incorporate recent findings linking dentate spikes to flexible object location memory processing (Dupret et al., Neuron 2024), which further support the idea that transient DG network states may contribute to memory resolution and spatial discrimination.

The text added to the Discussion is the following:

*“While our results show that reducing PV-mediated inhibition enhances fine spatial discrimination, an important question is how such modulation may be flexibly achieved under physiological conditions in the intact DG. PV interneuron activity is embedded within a broader inhibitory network that includes somatostatin (SOM)-expressing interneurons and disinhibitory VIP-expressing cells, whose coordinated activity is dynamically regulated by behavioral state and local circuit interactions (Turi et al, 2019; Pi et al, 2013). In addition, PV^+^ interneurons dynamics are influenced by neuromodulatory inputs and activity-dependent plasticity within inhibitory circuits (Hattori et al, 2017; Kullmann & Lamsa, 2007; Estarellas et al, 2026). Recent work suggests that novelty processing in the DG is associated with transient suppression of PV^+^ interneuron activity (Hainmueller et al, 2024), supporting the idea that inhibitory tone in DG can be flexibly adjusted during memory encoding. In this context, recent findings showing that dentate spikes support offline hippocampal reactivation and flexible object-location memory (McHugh et al, 2024) raise the possibility that transient DG network states, potentially involving rapid shifts in inhibitory dynamics, contribute to the encoding and discrimination of highly similar spatial representations. Such transient reductions in PV-mediated inhibition could provide a physiological mechanism for temporarily shifting the DG toward a high-sensitivity state, thereby facilitating the separation of overlapping experiences when fine discrimination is required. Conversely, restoration of inhibitory tone may help preserve representational consistency once memories have been encoded.”*

Pi H-J, Hangya B, Kvitsiani D, Sanders JI, Huang ZJ, Kepecs A (2013). Cortical interneurons that specialize in disinhibitory control. Nature 503(7477):521-524. <https://doi.org/10.1038/nature12676>

Turi GF, Li W-K, Chavlis S, Pandi I, O’Hare J, Priestley JB, Grosmark AD, Liao Z, Ladow M, Zhang JF, Zemelman BV, Poirazi P, Losonczy A (2019). Vasoactive Intestinal Polypeptide-Expressing Interneurons in the Hippocampus Support Goal-Oriented Spatial Learning. Neuron 101(6):1150-1165.e8. <https://doi.org/10.1016/j.neuron.2019.01.009>

Hainmueller T, Cazala A, Huang L-W, Bartos M (2024). Subfield-specific interneuron circuits govern the hippocampal response to novelty in male mice. Nat Commun 15:714. <https://doi.org/10.1038/s41467-024-44882-3>

Estarellas C, Álvarez-Salvado E, Pérez-Cervera L, Caramés JM, Pérez-Montoyo E, López-Madrona VJ, Garcia-Hernandez R, Mirasso CR, Canals S (2026). Synaptic plasticity in the perforant pathway drives inhibitory reorganization enhancing dentate gyrus functionality. iScience 29(3):114878. <https://doi.org/10.1016/j.isci.2026.114878>

Kullmann D & Lamsa K (2007). Long-term synaptic plasticity in hippocampal interneurons. *Nat Rev Neurosci* 8, 687-699. <https://doi.org/10.1038/nrn2207>

Hattori R, Kuchibhotla K, Froemke RC, Komiyaka T (2017). Functions and dysfunctions of neocortical inhibitory neuron subtypes. Nat Neurosci 20, 1199-1208. <https://doi.org/10.1038/nn.4619>

McHugh SB, Lopes-dos-Santos V, Castelli M, Gava GP, Thompson SE, Tam SKE, Hartwich K, Perry B, Toth R, Denison T, Sharott A, Dupret D (2024). Offline hippocampal reactivation during dentate spikes supports flexible memory. Neuron 112(22):p3768-3781. <https://doi.org/10.1016/j.neuron.2024.08.022>

**Finally, would you expect a different mechanism to be at play in spatial discrimination tasks that are DG dependent but don't involve novelty? (E.g. reference memory tasks that require fine discrimination?).**

We would not necessarily expect a completely different mechanism to operate in DG-dependent spatial discrimination tasks that do not explicitly involve novelty. Rather, we would expect the same general principle to apply across tasks in which similar spatial representations must be separated: the regulation of a sensitivity–consistency balance by inhibitory tone. In novelty-based tasks such as NOL, reduced PV-mediated inhibition may facilitate the detection and encoding of subtle changes by shifting the DG toward a high-sensitivity state. In reference memory tasks requiring fine discrimination, a similar shift could enhance the resolution of overlapping spatial representations, even in the absence of explicit novelty. However, the upstream signals that regulate inhibitory tone, as well as the behavioral readouts, may differ. In such tasks, inhibitory modulation may be driven less by novelty-related signals and more by task demands related to spatial precision, learned cue–goal associations, or retrieval under interference.

We added the following text to the Discussion:

*Although the present study focused on novelty-based object-location memory, the proposed mechanism is unlikely to be restricted to novelty processing. DG-dependent reference memory tasks requiring fine spatial discrimination also depend on the separation of overlapping spatial representations. We therefore expect PV-mediated inhibition to regulate a similar sensitivity–consistency balance in those tasks, although the upstream signals controlling inhibitory tone may differ from those engaged during novelty detection.*

**Reviewer #3: "A sensitivity-consistency trade-off in memory formation regulated by dentate gyrus inhibition" - Pérez-Montoyo et al., 2025**

**Summary:
 This paper explores a tradeoff between "sensitivity" and "consistency" in episodic memory, regulated by perisomatic inhibition of dentate gyrus (DG) granule cells (GC), mediated by parvalbumin-containing GABAergic interneurons (PV+). The authors modulated the activity of PV+ cells, using inhibitory and excitatory DREADDs, during different phases of several spatial/contextual memory tasks (encoding, consolidation, retrieval). Afferent synaptic input and dendritic integration were measured in vivo by the slope of EPSPs in the molecular layer (ML), and the output of DG was measured by the size of population spikes (PS) in the hilus. Activity of PV+ cells was measured by c-Fos staining. A computational model was introduced to account for several behavioral findings. The main conclusion is that there is an inverted-U-shaped relation between PV+ activity and memory behavior, such that low inhibition favors discrimination (e.g., sensitivity) at the cost of stability under low memory demand, whereas high inhibition favors stability (e.g., consistency) at the cost of discrimination under high memory demand.**

**General Comments:**

**The paper addresses an interesting and important question, using state of the art methods. One particular strength is the selective increase/decrease in PV+ cell activity, combined with examining the effects of those manipulations at multiple stages of the memory process. Another strength is the sophisticated set of behavioral tasks (but see also below). The writing is, in general, very clear and concise (but see also below). I have a few comments, mainly about how the interpretation of different experiments is explained in terms of the final conclusion. Most of these can be addressed by textual changes.**

**Specific Points:**

**1) Electrophysiology – The simultaneous use of two recording sites to separately measure EPSPs/dendritic integration in the molecular layer (ML) versus the DG "output" (PS) is very nice, and obviously an important control. However, no high-resolution traces of EPSPs or PS are shown. Fig1a, top, is useful for orientation but too small for close inspection, making it difficult to truly judge EPSP/PS separation. Sometimes ML EPSPs can be contaminated by PS if current sources/sinks are not well separated. Such an artifact could be ruled out by close visual inspection. Please add higher resolution traces to Fig 1, clearly showing the morphologies of EPSPs in the ML and PS in the hilus. It would also be very helpful to see high-res example traces of EPSPs/PS pre/post CNO and pre/post LTP.**

Thank you for pointing this out. We have added the requested high resolution traces to Fig. 1.

**Another issue is why the PS was measured in the hilus, where cell bodies and axons of multiple "local" interneuron types reside, including multiple inhibitory types as well as excitatory mossy cells. Most of these cell types would be driven by GC firing, but some would also be driven by direct perforant path (PP) stimulation. In contrast, the "output" of DG mainly arises from GCs alone. Why was the PS not measured directly in the GC layer instead? How are the authors sure that the hilar PS accurately reflects DG "output" (from GCs) and not also local interneuron firing? Some discussion of this possible artifact would be helpful.**

Traditionally, the population spike evoked by perforant path stimulation has often been recorded in the hilus, where its amplitude is particularly large and reproducible despite the fact that granule cell somata are located in the granule cell layer. This apparent paradox can be explained by the specific cytoarchitectonic and electrical organization of the dentate gyrus. Hilar field responses evoked by perforant path stimulation are large, robust, and highly reproducible. Importantly, previous experimental and modeling work has shown that the cytoarchitectonic organization of the dentate gyrus strongly favors the generation of large positive LFPs in the hilus. In particular, Fernández-Ruiz et al. (2013) demonstrated that the polarized dendritic arbor of granule cells, together with the curved bilaminar organization of the dentate gyrus, causes granule-cell-associated currents to spatially cluster in the hilus and to be volume-conducted away from the granule cell domains. In their model and recordings, large positive hilar LFPs required synchronous activation of granule cells in both blades of the dentate gyrus, whereas even mild reductions in spatial synchrony markedly reduced the distant hilar signal.

Thus, although local hilar neurons may contribute to the recorded response, the large, short-latency positive hilar population spike evoked by perforant path stimulation is expected to primarily reflect the synchronous recruitment of the granule cell population and its associated current geometry, rather than independent firing of heterogeneous hilar interneurons or mossy cells. This interpretation is further supported by the temporal profile of the response: the quantified population spike corresponds to the early, sharply time-locked component following perforant path stimulation, which is more consistent with synchronized granule cell recruitment than with heterogeneous, polysynaptic activation of local hilar populations.

Fernández-Ruiz A, Muñoz S, Sancho M, Makarova J, Makarov VA, Herreras O (2013). Cytoarchitectonic and Dynamic Origins of Giant Positive Local Field Potentials in the Dentate Gyrus. J Neurosci. 3 (39) 15518-15532; <https://doi.org/10.1523/JNEUROSCI.0338-13.2013>

**2) Behavioral Tasks – The authors have used a battery of several related, but not identical, behavioral tasks involving Novel Object Location (e.g., Simple NOL, Sequential NOL and an entirely new Multi-Arena NOL). Some of these (Simple and Sequential NOL) seem to probe the same sort of spatial memory for object location, but they differ along dimensions of task complexity and timing of the different task phases. The Multi-Arena NOL task differs along even more dimensions, including arena geometry, contextual cues, object identity, task complexity and phase timing. In my opinion, all of these tasks are useful and the experiments/data appear well-executed and reliable.**

**However, it seems that the authors are using results from different tasks to support entirely different parts of their final conclusion (i.e., tradeoff between sensitivity and consistency). The Simple and Sequential NOL are interpreted as supporting the idea that reduced PV+ activity favors "sensitivity" (enhanced discrimination), whereas the Multi-Arena NOL is interpreted as supporting the idea that reduced PV+ activity favors "consistency". Thus, the data supporting the former idea come from a very different sort of task than those supporting the latter idea. Some discussion of this apparent mismatch in methods/interpretations would be helpful.**

**It is also not entirely clear to me how the computational model specifically simulates any one of these individual tasks. Perhaps I'm mistaken, but the model doesn't seem to contain any explicit quantities that *separately* represent context, object identity or phase timing, etc. But I appreciate the authors' Discussion of the complexity of the problem and the advantages of a simple model with a simple conceptual interpretation.**

We have addressed the different points raised by the reviewer below.

**In summary, I think the paper would benefit from a clearer discussion of the following questions:**

**a) How do the different tasks relate to each other? Which tasks *specifically* support the idea that reduced PV+ activity favors "sensitivity" and which ones *specifically* support the idea that reduced PV+ activity favors "consistency"?**

We thank the reviewer for raising this important point. We agree that the different NOL-based paradigms used in the study differ in task structure, temporal organization and cognitive demand. However, they are closely related in that all of them rely on novel object–location/context associations. Our rationale for using complementary versions of the NOL paradigm was to impose different levels of demand on hippocampal processing and thereby assess distinct behavioral consequences of PV+ interneuron modulation.

Importantly, we do not interpret reduced PV+ activity as favoring “sensitivity” in one task and “consistency” in another as two independent or contradictory effects. Rather, our interpretation is that reducing PV+ activity shifts the system toward higher sensitivity. Depending on task demand, this shift can lead either to improved spatial discrimination or to reduced consistency/stability of the resulting representation.

Specifically, the standard NOL task, involving one object–location association with a 15 cm displacement, was used to determine whether PV+ interneuron modulation affects spatial memory formation and to define the temporal window in which this manipulation influences behavior. This experiment showed that PV+ modulation selectively affected memory encoding, but not consolidation or retrieval. In this task, decreasing PV+ activity increased discrimination, whereas increasing PV+ activity reduced discrimination.

The sequential NOL task was then designed to more directly assess the resolution of spatial discrimination. In this paradigm, animals first faced a more demanding discrimination condition, with objects separated by 10 cm while keeping the other main task parameters constant, followed by a later displacement to the standard 15 cm position. Under these conditions, PV+ inhibition led to an increase in discrimination, relative to the unmodulated control condition. Thus, the standard and sequential NOL experiments specifically support the conclusion that reduced PV^+^ activity enhances sensitivity and improves discrimination when task demands remain within a range in which increased sensitivity is beneficial.

Finally, the multi-arena NOL task introduced increased contextual and representational demands by requiring animals to process several object–context/location associations consecutively. This task was designed on the basis of the computational model, which suggested a tension between sensitivity and consistency as cognitive demand increases. In this framework, the multi-arena NOL experiment specifically tests the behavioral consequence of reduced PV^+^ activity under higher representational load. The results are therefore not interpreted as contradicting the sensitivity effect observed in the standard and sequential NOL tasks, but as revealing its cost: when demand is higher, a shift toward increased sensitivity can compromise the consistency or stability of the representation.

We have now clarified this logic in the manuscript. In summary, the standard and sequential NOL tasks support the conclusion that reduced PV^+^ activity favors sensitivity and spatial discrimination, whereas the multi-arena NOL task supports the conclusion that this increase in sensitivity can lead to a reduced performance under higher cognitive/representational demand. Thus, the apparent mismatch across tasks reflects the intended use of complementary paradigms to probe different points of the same sensitivity–consistency trade-off.

We have added this text to the Discussion:

*The different NOL-based paradigms used in this study were designed to probe complementary aspects of hippocampal-dependent memory processing under progressively increasing cognitive demands. Using the standard NOL task, we first established that PV^+^ interneuron modulation selectively affects memory encoding, without altering consolidation or retrieval processes. Building on this observation, we developed a sequential NOL paradigm to determine whether reduced PV-mediated inhibition altered the resolution of spatial discrimination. In this task, PV-Gi animals detected smaller spatial displacements than controls, indicating enhanced capacity to discriminate subtle spatial changes. We then asked whether this increased discrimination capacity carried a cost under conditions requiring the encoding and maintenance of multiple memory representations. To address this, we designed the multi-arena NOL task, which introduced increased contextual interference and representational demands through multiple object-location associations across distinct environments. Under these conditions, reduced PV-mediated inhibition impaired discrimination. Thus, while the standard and sequential NOL tasks indicate that reduced PV^+^ activity enhances discrimination when task demands are low to moderate, the multi-arena task reveals a performance cost when associative and interference demands increase. Together, these findings suggest that PV-dependent inhibitory dynamics contribute to balancing the sensitivity and consistency continuum.*

In order to relate the different variables visually, we have prepared the following figure only for review purposes, unless otherwise requested:

**Figure R4. Schematic representation of the load-dependent sensitivity–consistency trade-off.** The figure illustrates the proposed relationship between discrimination performance and the balance between consistency and sensitivity as a function of inhibitory tone. Three curves represent different levels of memory load: low load (light grey), medium load (grey), and high load (black). For each load, optimal discrimination occurs at a different point along the consistency–sensitivity axis, indicated by asterisks. As memory load increases, maximal discrimination decreases and the optimal operating point shifts toward a more consistent, inhibition-dominated state. The vertical dashed lines illustrate two experimental conditions. The orange line represents reduced inhibition, comparable to the PV-Gi condition, which enhances discrimination under low-load conditions but impairs discrimination when memory load is high. Conversely, the blue line represents increased inhibition, comparable to the PV-Gq condition, which favors consistency but reduces discrimination when fine sensitivity is required. Thus, as observed experimentally, disinhibition is predicted to have a stronger detrimental effect under high-load conditions, whereas its effect can be beneficial when memory load is low and increased sensitivity supports discrimination.

**b) What are the features of each task that might lead to one conclusion versus the other conclusion? It seems that the "sensitivity" idea relies on the simplest tasks (simple & sequential NOL) whereas the "consistency" argument relies on the most complex task (Multi-Arena NOL). The authors do indeed emphasize the idea that memory load is important. Nevertheless, I believe the average reader would benefit greatly from a brief, clear and concise Discussion of the task differences and how they relate to different parts of the final conclusion.**

We agree with the reviewer in that a more detailed explanation of the relevant features of each task was needed. We have added text to the discussion (see answer to point a above).

**c) How do parameters of the computational model map onto various aspects of the behavioral tasks? How are complex experimental parameters, such as context, object identity, etc, encoded in the model (if at all)? And if not, how/why should the reader interpret the model as being representative of the experimental results?**

We thank the reviewer for this insightful comment. The computational model does not explicitly represent task-specific variables such as contextual identity, object identity, arena geometry, or phase structure. Thus, there is no one-to-one mapping between these experimental parameters and individual parameters of the model. Our goal was not to reproduce each behavioral task in detail, but to capture a shared computational principle across them: how changes in PV-mediated inhibition influence the formation, discrimination, and stability of hippocampal-like memory representations.

For this reason, the model was deliberately formulated at an abstract level, in which memories are encoded as distributed representations without explicit contextual labels or task-specific procedural structure. This simplification allowed us to isolate the contribution of inhibitory dynamics to representational sensitivity and consistency, without introducing additional assumptions about how each behavioral variable should be encoded. In this sense, the model should be interpreted as a conceptual and mechanistic framework rather than as a detailed simulation of the individual behavioral paradigms.

Importantly, the model was useful in identifying a trade-off between sensitivity and consistency. Reduced inhibition increased the sensitivity of memory representations, which is consistent with the enhanced discrimination observed in the standard and sequential NOL tasks. However, the same shift also reduced representational consistency under conditions of higher load, providing a framework to interpret the impairment observed in the multi-arena NOL task. We have now clarified the scope and limitations of the model in the Discussion:.

*…“Moreover, the model does not explicitly represent task-specific variables such as contextual identity, object identity, arena geometry, or phase structure, which differ across the behavioral paradigms employed experimentally. Instead, memories are encoded as distributed representations without explicit contextual labels or procedural features.“...*

**Overall, I think the average reader would benefit from some *brief* discussion of the questions above. Especially the difference between the simple tasks that support the sensitivity idea versus the very complex task that supports the consistency idea.**

We thank the reviewer for this important question. The parameters of the computational model were not designed to map individually onto specific experimental variables such as context, object identity, or task phase structure. Instead, the model operates at a more abstract level, in which these task-specific elements are not explicitly represented. Rather than aiming to reproduce the detailed structure of each behavioral paradigm, the model was designed to capture a shared computational process across tasks, namely the level of PV-mediated inhibitory control and its impact on the formation and discrimination of memory representations. In this framework, experimental manipulations across different tasks can be interpreted as probing different behavioral expressions of changes in this underlying circuit-level mechanism, rather than as direct instantiations of model parameters.

We have clarified this point in the revised manuscript to better explain how the model should be interpreted (see answers above).

**3) Concepts and Nomenclature - I found a number of concepts and associated nomenclature in the text to be a bit hard to follow, given the common nomenclature in the literature about DG and hippocampal processing. Some brief clarification in Introduction and Discussion would be very helpful.**

**a) The authors focus on the concepts of "sensitivity" and "consistency". Sometimes "sensitivity" seems to be used interchangeably with "flexibility". Please clearly explain the relation/difference between these two words/concepts. Similarly, sometimes "consistency" seems to be used interchangeably with "stability". Again, please clarify the relation/difference between these two words/concepts.**

We thank the reviewer for highlighting this important conceptual issue. We agree that the distinction between these terms was not always sufficiently clear in the original manuscript. We have now revised the text to define them more explicitly and to use them more consistently throughout the manuscript.

In the revised version, we use “sensitivity” and “consistency” to refer to operational states of the network. Specifically, we define sensitivity as the network’s capacity to detect small differences in inputs and generate distinct neural representations. Conversely, we define consistency as the network’s tendency to maintain a reliable activity pattern despite minor input variations or noise.

We use “flexibility” and “stability” to refer to broader functional attributes of memory that can emerge from these network properties. Thus, increased network sensitivity can support memory flexibility, understood as the capacity to update or form distinct memory traces when relevant environmental changes occur. In contrast, increased network consistency can support memory stability, understood as the capacity of a memory trace to resist interference over time.

Finally, we reserve “discrimination” and “resistance to interference” for the behavioral manifestations assessed in our experiments. In this framework, the behavioral tasks do not directly measure sensitivity or consistency at the network level, but provide behavioral readouts from which these network properties can be inferred. We have now clarified these definitions in the Introduction and carefully revised the manuscript to avoid using sensitivity/flexibility or consistency/stability interchangeably.

**b) In the common nomenclature, the concepts of "pattern separation" and "pattern completion" are used to describe separate aspects of episodic memory. The authors refer to "pattern separation" a few times, in the context of "discrimination index". Do they mean that "pattern separation", "discrimination" and "sensitivity" refer to the same sort of thing (ability to tell apart similar episodes)? Some explanation of the relation between these words would be very helpful. They have shown that their results mainly apply to the encoding phase, so questions about "pattern completion" are perhaps not relevant for this study. Nevertheless, I believe that the average reader would benefit from a brief, clear explanation of how these different concepts/words are related. Ideally, this should be done early in the paper (Intro) rather than later (Discussion).**

We thank the reviewer for raising this important point. No, we do not use “pattern separation”, “discrimination”, and “sensitivity” as equivalent terms. We use “discrimination” as the behavioral readout of pattern separation. Pattern separation refers to the computational process by which similar input patterns are transformed into more distinct neural representations at the network level. When this process is effective, animals are expected to better discriminate between similar experiences or object–location configurations, which is what we measure behaviorally through the discrimination index.

The relationship with sensitivity is more nuanced. In our framework, sensitivity refers to the network’s capacity to respond to small differences in input. Higher sensitivity can favor pattern separation and behavioral discrimination when memory load remains within a range in which subtle differences can be encoded as distinct representations. This is consistent with the improved discrimination observed in the standard and sequential NOL tasks after reducing PV+ activity. However, our results and model suggest that this relationship is not linear or universally beneficial. Under higher memory load, increased sensitivity may reduce representational consistency, generating patterns that are less stable and therefore more difficult to separate reliably across experiences. In that situation, discrimination can be impaired, as observed in the multi-arena NOL task.

Thus, discrimination is the behavioral measure, pattern separation is the underlying computational operation, and sensitivity is a network property that can support pattern separation depending on task demand and memory load. We now bring these points in the Introduction.

Regarding pattern completion, we agree with the reviewer that it is not central to the present study. Pattern completion is generally associated with recall from partial cues and is classically linked to CA3, in part because of its dense recurrent connectivity. Our study focused on DG-dependent encoding-related processes and did not examine how pattern completion interacts with the sensitivity–consistency trade-off in the DG-CA3 circuit. We agree that this is an important question and one that could be addressed in future work.

**4) Initial Representation versus Memory - The authors show that, under their protocols, the PV+ manipulation only matters if delivered during the encoding phase of the task, not during consolidation or retrieval (at least for simple NOL). But their final measures and conclusions are typically based on measurements during the retrieval phase. There may be a disconnect in reasoning here. Is it possible that all of their results about "memory" are not actually about memory, but rather about how PV+ activity alters the *initial* representation that was present in DG when the initial episode was presented for the first time?**

**For example, I can imagine that lower inhibition during the encoding phase would allow more GC firing across different "patterns", allowing for greater "sensitivity" to different patterns (i.e., more different patterns get *represented*, altogether). But I can also imagine that higher inhibition would narrow down the number of different patterns represented (i.e., fewer possible patterns can get *represented* at all).**

**Neither example above has anything to do with "memory", but rather what was *represented* in the DG during the initial presentation of the stimulus. Can the authors please discuss this issue? Does their study actually concern "memory" or does it actually address "representation" of initial patterns in the first place? Their finding that their manipulations only matter during "encoding" would seem to favor affecting the initial representation, rather than downstream memory processes. A brief discussion (or refutation) of this criticism would be helpful. In Discussion.**

We thank the reviewer for raising this important conceptual point. We agree that our results indicate that PV+ interneuron modulation acts primarily during encoding, and therefore most likely affects the initial representation when the object–location episode is first experienced. We do not view this as separate from memory, but rather as an early step in memory formation. The representation generated during encoding provides the substrate upon which subsequent consolidation and later retrieval operate.

In this sense, our study does not suggest that PV+ activity modulates memory expression at retrieval, nor that it directly alters downstream consolidation processes under the conditions tested in the simple NOL task. Instead, our data support the interpretation that PV-mediated inhibition shapes the structure of the initial representation during encoding, and that this altered representation is later revealed behaviorally as a change in memory-guided discrimination during retrieval.

This distinction is important. Reduced PV+ activity may increase the number or diversity of input patterns represented by granule cells, thereby increasing sensitivity to subtle differences between object–location configurations. Conversely, increased PV+ activity may restrict the range of represented patterns, reducing sensitivity but potentially favoring more consistent representations.

We have now clarified this point in the Discussion. Specifically, we state that our findings concern memory formation through the modulation of encoding-stage representations. Because the final behavioral readout is obtained during retrieval, we use the term memory to refer to the persistence and later behavioral expression of these encoded representations. However, we agree with the reviewer that the primary locus of PV+ modulation in our experiments is the initial representation formed during encoding.

We added the following text to the Discussion:

*“Notably, although PV^+^ activity was manipulated across encoding, consolidation, and retrieval phases, behavioral effects were observed only when the manipulation occurred during encoding. This phase specificity indicates that PV-mediated inhibition primarily shapes the initial hippocampal representation formed during the first experience, thereby influencing how the episode is encoded and later expressed during retrieval.”*
