# Supplementary material for: Inhibitory tone in the dentate gyrus dynamically prioritizes memory flexibility or stability by tuning a sensitivity-consistency continuum
Source: PLoS Biol. 2026 Aug 18;24(8):e3003956. doi: 10.1371/journal.pbio.3003956 (PMC13506102; doi:10.1371/journal.pbio.3003956)
Supplement: Peer review history

# Editor report: Decision Letter 0

**DOI:** 10.1371/journal.pbio.3003956.r001

**Authors:** Christian Schnell, PhD

8 Oct 2025

Dear Dr Marcos,

Thank you for submitting your manuscript entitled "A sensitivity-consistency trade-off in memory formation regulated by dentate gyrus inhibition" for consideration as a *Short Report* by PLOS Biology.

Your manuscript has now been evaluated by the PLOS Biology editorial staff as well as by an academic editor with relevant expertise and I am writing to let you know that we would like to send your submission out for external peer review.

However, before we can send your manuscript to reviewers, we need you to complete your submission by providing the metadata that is required for full assessment. To this end, please login to Editorial Manager where you will find the paper in the 'Submissions Needing Revisions' folder on your homepage. Please click 'Revise Submission' from the Action Links and complete all additional questions in the submission questionnaire.

Once your full submission is complete, your paper will undergo a series of checks in preparation for peer review. After your manuscript has passed the checks it will be sent out for review. To provide the metadata for your submission, please Login to Editorial Manager (https://www.editorialmanager.com/pbiology) within two working days, i.e. by Oct 10 2025 11:59PM.

If your manuscript has been previously peer-reviewed at another journal, PLOS Biology is willing to work with those reviews in order to avoid re-starting the process. Submission of the previous reviews is entirely optional and our ability to use them effectively will depend on the willingness of the previous journal to confirm the content of the reports and share the reviewer identities. Please note that we reserve the right to invite additional reviewers if we consider that additional/independent reviewers are needed, although we aim to avoid this as far as possible. In our experience, working with previous reviews does save time.

If you would like us to consider previous reviewer reports, please edit your cover letter to let us know and include the name of the journal where the work was previously considered and the manuscript ID it was given. In addition, please upload a response to the reviews as a 'Prior Peer Review' file type, which should include the reports in full and a point-by-point reply detailing how you have or plan to address the reviewers' concerns.

During the process of completing your manuscript submission, you will be invited to opt-in to posting your pre-review manuscript as a bioRxiv preprint. Visit http://journals.plos.org/plosbiology/s/preprints for full details. If you consent to posting your current manuscript as a preprint, please upload a single Preprint PDF.

Feel free to email us at plosbiology@plos.org if you have any queries relating to your submission.

Kind regards,

Christian

Christian Schnell, PhD

Senior Editor

PLOS Biology

cschnell@plos.org

# Peer review: Decision Letter 1

**DOI:** 10.1371/journal.pbio.3003956.r002

**Authors:** Christian Schnell, PhD

4 Dec 2025

Dear Dr Marcos,

Thank you for your patience while your manuscript "A sensitivity-consistency trade-off in memory formation regulated by dentate gyrus inhibition" was peer-reviewed at PLOS Biology. It has now been evaluated by the PLOS Biology editors, an Academic Editor with relevant expertise, and by several independent reviewers.

In light of the reviews, which you will find at the end of this email, we would like to invite you to revise the work to thoroughly address the reviewers' reports.

As you will see below, Reviewer 2 and Reviewer 3 are very supportive and mostly request textual clarifications. Reviewer 1, however, highlights some important methodological issues that limit the conclusions that can currently be drawn from the data. They suggest a number of experiments that will strengthen the experimental support for the claims and conclusions from your study and we strongly encourage you to provide this data in your revised manuscript.

Given the extent of revision needed, we cannot make a decision about publication until we have seen the revised manuscript and your response to the reviewers' comments. Your revised manuscript is likely to be sent for further evaluation by all or a subset of the reviewers.

In addition to these revisions, you will need to complete some formatting changes, which you will receive in a follow up email. A member of our team will be in touch with a set of requests shortly.

We expect to receive your revised manuscript within 3 months. Please email us (plosbiology@plos.org) if you have any questions or concerns, or would like to request an extension.

At this stage, your manuscript remains formally under active consideration at our journal; please notify us by email if you do not intend to submit a revision so that we may withdraw it.

**IMPORTANT - SUBMITTING YOUR REVISION**

Your revisions should address the specific points made by each reviewer. Please submit the following files along with your revised manuscript:

1. A 'Response to Reviewers' file - this should detail your responses to the editorial requests, present a point-by-point response to all of the reviewers' comments, and indicate the changes made to the manuscript.

*NOTE: In your point-by-point response to the reviewers, please provide the full context of each review. Do not selectively quote paragraphs or sentences to reply to. The entire set of reviewer comments should be present in full and each specific point should be responded to individually, point by point.

You should also cite any additional relevant literature that has been published since the original submission and mention any additional citations in your response.

2. In addition to a clean copy of the manuscript, please also upload a 'track-changes' version of your manuscript that specifies the edits made. This should be uploaded as a "Revised Article with Changes Highlighted" file type.

*Re-submission Checklist*

When you are ready to resubmit your revised manuscript, please refer to this re-submission checklist: https://plos.io/Biology_Checklist

To submit a revised version of your manuscript, please go to https://www.editorialmanager.com/pbiology/ and log in as an Author. Click the link labelled 'Submissions Needing Revision' where you will find your submission record.

Please make sure to read the following important policies and guidelines while preparing your revision:

*Published Peer Review*

Please note while forming your response, if your article is accepted, you may have the opportunity to make the peer review history publicly available. The record will include editor decision letters (with reviews) and your responses to reviewer comments. If eligible, we will contact you to opt in or out. Please see here for more details:

https://blogs.plos.org/plos/2019/05/plos-journals-now-open-for-published-peer-review/

*PLOS Data Policy*

Please note that as a condition of publication PLOS' data policy (http://journals.plos.org/plosbiology/s/data-availability) requires that you make available all data used to draw the conclusions arrived at in your manuscript. If you have not already done so, you must include any data used in your manuscript either in appropriate repositories, within the body of the manuscript, or as supporting information (N.B. this includes any numerical values that were used to generate graphs, histograms etc.). For an example see here: http://www.plosbiology.org/article/info%3Adoi%2F10.1371%2Fjournal.pbio.1001908#s5

*Blot and Gel Data Policy*

We require the original, uncropped and minimally adjusted images supporting all blot and gel results reported in an article's figures or Supporting Information files. We will require these files before a manuscript can be accepted so please prepare them now, if you have not already uploaded them. Please carefully read our guidelines for how to prepare and upload this data: https://journals.plos.org/plosbiology/s/figures#loc-blot-and-gel-reporting-requirements

*Protocols deposition*

To enhance the reproducibility of your results, we recommend that if applicable you deposit your laboratory protocols in protocols.io, where a protocol can be assigned its own identifier (DOI) such that it can be cited independently in the future. Additionally, PLOS ONE offers an option for publishing peer-reviewed Lab Protocol articles, which describe protocols hosted on protocols.io. Read more information on sharing protocols at https://plos.org/protocols?utm_medium=editorial-email&utm_source=authorletters&utm_campaign=protocols

Thank you again for your submission to our journal. We hope that our editorial process has been constructive thus far, and we welcome your feedback at any time. Please don't hesitate to contact us if you have any questions or comments.

Sincerely,

Christian

Christian Schnell, PhD

Senior Editor

PLOS Biology

cschnell@plos.org

------------------------------------

REVIEWS:

Reviewer #1: Pérez-Montoyo et al., describe a series of physiological, behavioral, and computational experiments to elucidate the role of hippocampal parvalbumin-expressing interneuron in object-location learning. The authors focus on an important question and have made a substantial effort to address it experimentally. Unfortunately, there are several limitations in the interpretability of their data, in part due to inherent technical limitations of the approach and the experimental design. There are also several key conclusions which are not sufficiently supported by the data presented by the authors in my opinion.

Major points:

1.As shown on Figure S1, the viral constructs used by the authors affect PV interneurons in both the DG and CA3. This problem is not unique to their study (see e.g. Aery Jones et al., 2021, Cell Reports) but the assumption that the manipulation affects predominantly the DG is the only thing that links the experiment to the model here and is therefore critical.

2.There are several assumptions inherent to the model about how sparsity or overlap of granule cell activity relates to performance in discrimination tasks that have no link to the experiments. The bare minimum the authors could do is to quantify the cfos expression in GCs in addition to the cfos expression in PV interneurons, similar to e.g. Stefanelli et al., 2016, Neuron (which should also be discussed as they perform extremely similar experiments) or Chawla et al., 2005, Hippocampus. A measure of GC activity through calcium imaging or electrophysiology during interneuron manipulations could decisively strengthen a potential link to the model and help discriminate effects of granule cells from those of mossy cells and CA3 pyramidal cells (see also point 1).

3.On page 4, line 3, the authors claim that "the impaired performance observed in PV-Gq animals likely reflects a reduction in discrimination capacity rather than a general deficit in spatial memory encoding". While stated here as a possibility, the authors later use this as a central tenet of their manuscript without ever backing it with direct evidence. To support this claim, they should show whether a version of this task with a larger displacement or a shorter interval to testing can be learned by the animals to show that the deficit is not related to a global impairment of spatial memory or decreased stability of a formed memory trace.

4.The outcome of the task in Fig 2f,g is ambiguous: One can equally well argue that the increased interest in the displaced object on the second trial on day 2 reflects learning of the 5 cm displacement compared to the first trial on that day one minute prior. The better comparator would be the original NOL task with 15 cm displacement and 24 hour interval.

5.For the task on Figure 4, it is unclear whether this reflects a deficit in context discrimination or primarily of increased workload as claimed by the authors. To distinguish these possibilities, we would need to know how animals in the different groups perform in a standard contextual conditioning task with a 24 hour interval.

Minor points

1.Figure 1d: There should be a comparison between the magnitude of LTP in the three groups. It appears that plasticity increases both with activation and inhibition of PV interneurons. It would be interesting to see if that difference is significant and I would encourage the authors to discuss it.

2.Fig S1: Should also show the fraction of DREADD expressing cells that are PV positive to rule out leak expression in non-target cells.

Reviewer #2: The manuscript by Pérez-Montoyo, María Caramés et al tackles an interesting question of how the memory system achieves sensitivity and consistency. I found these results to be very counterintuitive at first - especially the idea that reducing inhibition increases discrimination - so definitely a provocative study! The authors did an excellent job of walking me through their results and I especially appreciated the results from the computational mode.

Please address the following:

For the NOL encoding result, could the authors also report the total time exploring objects during the encoding phase (sample phase)? In other words, please exclude the possibility that the DI differences between the 3 groups during testing is not a result of reduced exploration during the sample phase. I see in the methods that the sample phase was terminated after 20 s of exploration of both objects or after 10 min in the arena.

In 2G, it seems that the control is not able to perform the task for the 10 cm displacement. Please consider results in (https://elifesciences.org/articles/65786), in which controls are sensitive to 10 cm displacement.

Along these lines, while your results indicate that PV disinhibition promotes discrimination - they don't help explain why fine discrimination is dependent on the dentate gyrus. (In fact, you don't observe discrimination of 10 cm in the intact case). Could you add to your discussion and speculate on how PV disinhibition may be flexibly realized in the DG? It could be useful to consider recent data about dentate spikes during NOL memory (Dupret).

Finally, would you expect a different mechanism to be at play in spatial discrimination tasks that are DG dependent but don't involve novelty? (E.g. reference memory tasks that require fine discrimination?)

Reviewer #3: "A sensitivity-consistency trade-off in memory formation regulated by dentate gyrus inhibition"

- Pérez-Montoyo et al., 2025

Summary:

This paper explores a tradeoff between "sensitivity" and "consistency" in episodic memory, regulated by perisomatic inhibition of dentate gyrus (DG) granule cells (GC), mediated by parvalbumin-containing GABAergic interneurons (PV+). The authors modulated the activity of PV+ cells, using inhibitory and excitatory DREADDs, during different phases of several spatial/contextual memory tasks (encoding, consolidation, retrieval). Afferent synaptic input and dendritic integration were measured in vivo by the slope of EPSPs in the molecular layer (ML), and the output of DG was measured by the size of population spikes (PS) in the hilus. Activity of PV+ cells was measured by c-Fos staining. A computational model was introduced to account for several behavioral findings. The main conclusion is that there is an inverted-U-shaped relation between PV+ activity and memory behavior, such that low inhibition favors discrimination (e.g., sensitivity) at the cost of stability under low memory demand, whereas high inhibition favors stability (e.g., consistency) at the cost of discrimination under high memory demand.

General Comments:

The paper addresses an interesting and important question, using state of the art methods. One particular strength is the selective increase/decrease in PV+ cell activity, combined with examining the effects of those manipulations at multiple stages of the memory process. Another strength is the sophisticated set of behavioral tasks (but see also below). The writing is, in general, very clear and concise (but see also below). I have a few comments, mainly about how the interpretation of different experiments is explained in terms of the final conclusion. Most of these can be addressed by textual changes.

Specific Points:

1) Electrophysiology -

The simultaneous use of two recording sites to separately measure EPSPs/dendritic integration in the molecular layer (ML) versus the DG "output" (PS) is very nice, and obviously an important control. However, no high-resolution traces of EPSPs or PS are shown. Fig1a, top, is useful for orientation but too small for close inspection, making it difficult to truly judge EPSP/PS separation. Sometimes ML EPSPs can be contaminated by PS if current sources/sinks are not well separated. Such an artifact could be ruled out by close visual inspection. Please add higher resolution traces to Fig 1, clearly showing the morphologies of EPSPs in the ML and PS in the hilus. It would also be very helpful to see high-res example traces of EPSPs/PS pre/post CNO and pre/post LTP.

Another issue is why the PS was measured in the hilus, where cell bodies and axons of multiple "local" interneuron types reside, including multiple inhibitory types as well as excitatory mossy cells. Most of these cell types would be driven by GC firing, but some would also be driven by direct perforant path (PP) stimulation. In contrast, the "output" of DG mainly arises from GCs alone. Why was the PS not measured directly in the GC layer instead? How are the authors sure that the hilar PS accurately reflects DG "output" (from GCs) and not also local interneuron firing? Some discussion of this posible artifact would be helpful.

2) Behavioral Tasks -

The authors have used a battery of several related, but not identical, behavioral tasks involving Novel Object Location (e.g., Simple NOL, Sequential NOL and an entirely new Multi-Arena NOL). Some of these (Simple and Sequential NOL) seem to probe the same sort of spatial memory for object location, but they differ along dimensions of task complexity and timing of the different task phases. The Multi-Arena NOL task differs along even more dimensions, including arena geometry, contextual cues, object identity, task complexity and phase timing. In my opinion, all of these tasks are useful and the experiments/data appear well-executed and reliable.

However, it seems that the authors are using results from different tasks to support entirely different parts of their final conclusion (i.e., tradeoff between sensitivity and consistency). The Simple and Sequential NOL are interpreted as supporting the idea that reduced PV+ activity favors "sensitivity" (enhanced discrimination), whereas the Multi-Arena NOL is interpreted as supporting the idea that reduced PV+ activity favors "consistency". Thus, the data supporting the former idea come from a very different sort of task than those supporting the latter idea. Some discussion of this apparent mismatch in methods/interpretations would be helpful.

It is also not entirely clear to me how the computational model specifically simulates any one of these individual tasks. Perhaps I'm mistaken, but the model doesn't seem to contain any explicit quantities that *separately* represent context, object identity or phase timing, etc. But I appreciate the authors' Discussion of the complexity of the problem and the advantages of a simple model with a simple conceptual interpretation.

In summary, I think the paper would benefit from a clearer discussion of the following questions:

a) How do the different tasks relate to each other? Which tasks *specifically* support the idea that reduced PV+ activity favors "sensitivity" and which ones *specifically* support the idea that reduced PV+ activity favors "consistency"?

b) What are the features of each task that might lead to one conclusion versus the other conclusion? It seems that the "sensitivity" idea relies on the simplest tasks (simple & sequential NOL) whereas the "consistency" argument relies on the most complex task (Multi-Arena NOL). The authors do indeed emphasize the idea that memory load is important. Nevertheless, I believe the average reader would benefit greatly from a brief, clear and concise Discussion of the task differences and how they relate to different parts of the final conclusion.

c) How do parameters of the computational model map onto various aspects of the behavioral tasks? How are complex experimental parameters, such as context, object identity, etc, encoded in the model (if at all)? And if not, how/why should the reader interpret the model as being representative of the experimental results?

Overall, I think the average reader would benefit from some *brief* discussion of the questions above. Especially the difference between the simple tasks that support the sensitivity idea versus the very complex task that supports the consistency idea.

3) Concepts and Nomenclature -

I found a number of concepts and associated nomenclature in the text to be a bit hard to follow, given the common nomenclature in the literature about DG and hippocampal processing. Some brief clarification in Introduction and Discussion would be very helpful.

a) The authors focus on the concepts of "sensitivity" and "consistency". Sometimes "sensitivity" seems to be used interchangeably with "flexibility". Please clearly explain the relation/difference between these two words/concepts. Similarly, sometimes "consistency" seems to be used interchangeably with "stability". Again, please clarify the relation/difference between these two words/concepts.

b) In the common nomenclature, the concepts of "pattern separation" and "pattern completion" are used to describe separate aspects of episodic memory. The authors refer to "pattern separation" a few times, in the context of "discrimination index". Do they mean that "pattern separation", "discrimination" and "sensitivity" refer to the same sort of thing (ability to tell apart similar episodes)? Some explanation of the relation between these words would be very helpful. They have shown that their results mainly apply to the encoding phase, so questions about "pattern completion" are perhaps not relevant for this study. Nevertheless, I believe that the average reader would benefit from a brief, clear explanation of how these different concepts/words are related. Ideally, this should be done early in the paper (Intro) rather than later (Discussion).

4) Initial Representation versus Memory -

The authors show that, under their protocols, the PV+ manipulation only matters if delivered during the encoding phase of the task, not during consolidation or retrieval (at least for simple NOL). But their final measures and conclusions are typically based on measurements during the retrieval phase. There may be a disconnect in reasoning here. Is it possible that all of their results about "memory" are not actually about memory, but rather about how PV+ activity alters the *initial* representation that was present in DG when the initial episode was presented for the first time?

For example, I can imagine that lower inhibition during the encoding phase would allow more GC firing across different "patterns", allowing for greater "sensitivity" to different patterns (i.e., more different patterns get *represented*, altogether). But I can also imagine that higher inhibition would narrow down the number of different patterns represented (i.e., fewer possible patterns can get *represented* at all).

Neither example above has anything to do with "memory", but rather what was *represented* in the DG during the initial presentation of the stimulus. Can the authors please discuss this issue? Does their study actually concern "memory" or does it actually address "representation" of initial patterns in the first place? Their finding that their manipulations only matter during "encoding" would seem to favor affecting the initial representation, rather than downstream memory processes. A brief discussion (or refutation) of this criticism would be helpful. In Discussion.

# Author comment: Author response to Decision Letter 2

**DOI:** 10.1371/journal.pbio.3003956.r003

17 Jun 2026

Attachment Submitted filename: Perez-Montoyo_responsetoREVIEWERS.docx

# Peer review: Decision Letter 2

**DOI:** 10.1371/journal.pbio.3003956.r004

**Authors:** Christian Schnell, PhD

17 Jul 2026

Dear Dr Marcos,

Thank you for your patience while we considered your revised manuscript "A sensitivity-consistency trade-off in memory formation regulated by dentate gyrus inhibition" for publication as a Research Article at PLOS Biology. This revised version of your manuscript has been evaluated by the PLOS Biology editors, the Academic Editor and two of the original reviewers.

Based on the reviews and on our Academic Editor's assessment of your revision, we are likely to accept this manuscript for publication, provided you satisfactorily address the remaining points raised by the reviewers and the following data and other policy-related requests:

* We would like to suggest a different title to improve its accessibility for our broad audience:

The dentate gyrus dynamically prioritizes flexibility or stability in memory formation depending on inhibitory tone

* Please add the links to the funding agencies in the Financial Disclosure statement in the manuscript details.

* DATA POLICY:

You may be aware of the PLOS Data Policy, which requires that all data be made available without restriction: http://journals.plos.org/plosbiology/s/data-availability. For more information, please also see this editorial: http://dx.doi.org/10.1371/journal.pbio.1001797

Note that we do not require all raw data. Rather, we ask that all individual quantitative observations that underlie the data summarized in the figures and results of your paper be made available in one of the following forms:

1) Supplementary files (e.g., excel). Please ensure that all data files are uploaded as 'Supporting Information' and are invariably referred to (in the manuscript, figure legends, and the Description field when uploading your files) using the following format verbatim: S1 Data, S2 Data, etc. Multiple panels of a single or even several figures can be included as multiple sheets in one excel file that is saved using exactly the following convention: S1_Data.xlsx (using an underscore).

2) Deposition in a publicly available repository. Please also provide the accession code or a reviewer link so that we may view your data before publication.

Regardless of the method selected, please ensure that you provide the individual numerical values that underlie the summary data displayed in the following figure panels as they are essential for readers to assess your analysis and to reproduce it: 1BCD, 2BCDEFH, 3E, 4BD, S1C, S2CD, S3BD and S4CE

NOTE: the numerical data provided should include all replicates AND the way in which the plotted mean and errors were derived (it should not present only the mean/average values).

Please also ensure that figure legends in your manuscript include information on where the underlying data can be found, and ensure your supplemental data file/s has a legend.

Please ensure that your Data Statement in the submission system accurately describes where your data can be found.

* CODE POLICY

Per journal policy, if you have generated any custom code during the course of this investigation, please make it available without restrictions. Please ensure that the code is sufficiently well documented and reusable, and that your Data Statement in the Editorial Manager submission system accurately describes where your code can be found. More information on our Code Policy, what and how to share can be found here: https://journals.plos.org/plosbiology/s/code-availability

Please note that we cannot accept sole deposition of code in GitHub, as this could be changed after publication. However, you can archive this version of your publicly available GitHub code to Zenodo. Once you do this, it will generate a DOI number, which you will need to provide in the Data Accessibility Statement (you are welcome to also provide the GitHub access information). See the process for doing this here: https://docs.github.com/en/repositories/archiving-a-github-repository/referencing-and-citing-content

As you address these items, please take this last chance to review your reference list to ensure that it is complete and correct. If you have cited papers that have been retracted, please include the rationale for doing so in the manuscript text, or remove these references and replace them with relevant current references. Any changes to the reference list should be mentioned in the cover letter that accompanies your revised manuscript.

In addition to these revisions, you may need to complete some formatting changes, which you will receive in a follow up email. A member of our team will be in touch with a set of requests shortly. If you do not receive a separate email within a few days, please assume that checks have been completed, and no additional changes are required.

We expect to receive your revised manuscript within two weeks.

To submit your revision, please go to https://www.editorialmanager.com/pbiology/ and log in as an Author. Click the link labelled 'Submissions Needing Revision' to find your submission record. Your revised submission must include the following:

- a cover letter that should detail your responses to any editorial requests, if applicable, and whether changes have been made to the reference list

- a Response to Reviewers file that provides a detailed response to the reviewers' comments (if applicable, if not applicable please do not delete your existing 'Response to Reviewers' file.)

- a track-changes file indicating any changes that you have made to the manuscript.

NOTE: If Supporting Information files are included with your article, note that these are not copyedited and will be published as they are submitted. Please ensure that these files are legible and of high quality (at least 300 dpi) in an easily accessible file format. For this reason, please be aware that any references listed in an SI file will not be indexed. For more information, see our Supporting Information guidelines:

https://journals.plos.org/plosbiology/s/supporting-information

*Published Peer Review History*

Please note that you may have the opportunity to make the peer review history publicly available. The record will include editor decision letters (with reviews) and your responses to reviewer comments. If eligible, we will contact you to opt in or out. Please see here for more details:

https://plos.org/published-peer-review-history/

*Press*

Should you, your institution's press office or the journal office choose to press release your paper, please ensure you have opted out of Early Article Posting on the submission form. We ask that you notify us as soon as possible if you or your institution is planning to press release the article.

*Protocols deposition*

To enhance the reproducibility of your results, we recommend that if applicable you deposit your laboratory protocols in protocols.io, where a protocol can be assigned its own identifier (DOI) such that it can be cited independently in the future. Additionally, PLOS ONE offers an option for publishing peer-reviewed Lab Protocol articles, which describe protocols hosted on protocols.io. Read more information on sharing protocols at https://plos.org/protocols?utm_medium=editorial-email&utm_source=authorletters&utm_campaign=protocols

Please do not hesitate to contact me should you have any questions.

Sincerely,

Christian

Christian Schnell, PhD

Senior Editor

cschnell@plos.org

PLOS Biology

------------------------------------------------------------------------

Reviewer remarks:

Reviewer #1: The authors addressed my concerns sufficiently.

Reviewer #3 (Mathew V Jones has signed his report): The authors have carefully, thoughtfully and thoroughly addressed my comments.

I now understand the logic and usefulness (and biological relevance) of this approach:

"For this reason, the model was deliberately formulated at an abstract level, in which memories are encoded as distributed representations without explicit contextual labels or task-specific procedural structure."

The figure provided in the authors' rebuttal "only for review purposes, unless otherwise requested" is a nice visual summary of a lot of complex experiments, analyses and reasoning. I encourage, but do not insist, the authors to find space to include this figure in the final draft, near Discussion. It helped me to grasp their overall point. I think it will help the average reader even more.

# Editor report: Decision Letter 3

**DOI:** 10.1371/journal.pbio.3003956.r005

**Authors:** Christian Schnell, PhD

4 Aug 2026

Dear Dr Marcos,

Thank you for the submission of your revised Research Article "Inhibitory tone in the dentate gyrus dynamically prioritizes memory flexibility or stability by tuning a sensitivity-consistency continuum" for publication in PLOS Biology. On behalf of my colleagues and the Academic Editor, Thomas Klausberger, I am pleased to say that we can in principle accept your manuscript for publication, provided you address any remaining formatting and reporting issues. These will be detailed in an email you should receive within 2-3 business days from our colleagues in the journal operations team; no action is required from you until then. Please note that we will not be able to formally accept your manuscript and schedule it for publication until you have completed any requested changes.

While you attend to those requests, please also ensure that you have listed the custom code in the Data Availability Statement and not only in the manuscript: "Custom MATLAB-based code developed in the lab (RodEx) is available at https://doi.org/10.20350/digitalCSIC/28955)"

Please take a minute to log into Editorial Manager at http://www.editorialmanager.com/pbiology/, click the "Update My Information" link at the top of the page, and update your user information to ensure an efficient production process.

PRESS

We frequently collaborate with press offices. If your institution or institutions have a press office, please notify them about your upcoming paper at this point, to enable them to help maximise its impact. If the press office is planning to promote your findings, we would be grateful if they could coordinate with biologypress@plos.org. If you have previously opted in to the early version process, we ask that you notify us immediately of any press plans so that we may opt out on your behalf.

We also ask that you take this opportunity to read our Embargo Policy regarding the discussion, promotion and media coverage of work that is yet to be published by PLOS. As your manuscript is not yet published, it is bound by the conditions of our Embargo Policy. Please be aware that this policy is in place both to ensure that any press coverage of your article is fully substantiated and to provide a direct link between such coverage and the published work. For full details of our Embargo Policy, please visit http://www.plos.org/about/media-inquiries/embargo-policy/.

Thank you again for choosing PLOS Biology for publication and supporting Open Access publishing. We look forward to publishing your study.

Sincerely,

Christian

Christian Schnell, PhD

Senior Editor

PLOS Biology

cschnell@plos.org
